# One and Two Dimensional Pulsed Electron Paramagnetic Resonance Studies of *in vivo* Vanadyl Coordination in Rat Kidney

**DOI:** 10.1155/S1565363303000062

**Published:** 2003

**Authors:** Sergei A. Dikanov, Barry D. Liboiron, Katherine H. Thompson, Erika Vera, Violet G. Yuen, John H. McNeill, Chris Orvig

**Affiliations:** 1 Illinois EPR Research Center and Department of Veterinary Clinical Medicine 190 MSB 506 S. Mathews Ave. University of Illinois at Urbana-Champaign Urbana IL 61801 USA; 2 Institute of Chemical Kinetics and Combustion Russian Academy of Sciences Novosibirsk 6300090 Russia; 3 Medicinal Inorganic Chemistry Group Department of Chemistry University of British Columbia 2036 Main Mall Vancouver BC V6T 1Z1 Canada; 4 Faculty of Pharmaceutical Sciences University of British Columbia 2146 East Mall Vancouver BC V6T 1Z3 Canada; 5 Department of Chemistry Stanford University Stanford CA 94305 USA

## Abstract

The biological fate of a chelated vanadium source is investigated by/n vivo spectroscopic methods to elucidate the chemical form in which the metal ion is accumulated. A pulsed electron paramagnetic resonance study of vanadyl ions in kidney tissue, taken from rats previously treated with bis(ethylmaltolato)oxovanadium(IV) (BEOV) in drinking water, is presented. A combined approach using stimulated echo (3-pulse) electron spin echo envelope modulation (ESEEM) and the two dimensional 4-pulse hyperfine sublevel correlation (HYSCORE) spectroscopies has shown that at least some of the VO^2+^ ions are involved in the coordination with nitrogen-containing ligands. From the experimental spectra, a 4N hyperfine coupling constant of 4.9 MHz and a quadrupole coupling constant of 0.6 + 0.04 MHz were determined, consistent with amine coordination of the vanadyl ions. Study of VO-histidine model complexes allowed for a determination of the percentage of nitrogen-coordinated VO^2+^ ions in the tissue sample that is found nitrogen-coordinated. By taking into account the bidentate nature of histidine coordination to VO^2+^ ions, a more accurate determination of this value is reported. The biological fate of chelated versus free (i.e. salts) vanadyl ion sources has been deduced by comparison to earlier reports. In contrast to its superior pharmacological efficacy over VOSO4, BEOV shares a remarkably similar biological fate after uptake into kidney tissue.


### One and Two Dimensional Pulsed Electron Paramagnetic Resonance Studies of *in vivo* Vanadyl Coordination in Rat Kidney

Sergei A. Dikanov,[1,2,*] Barry D. Liboiron,[3,+] Katherine H. Thompson,[3] Erika Vera,[4] Violet G. Yuen,[4] John H. McNeill[4] and Chris Orvig[3,*]

[1]*Illinois EPR Research Center and Department of Veterinary Clinical Medicine, 190 MSB, 506 S. Mathews Ave., University of Illinois at Urbana-Champaign, Urbana, IL 61801, U. S. A.*
[2]*Institute of Chemical Kinetics and Combustion, Russian Academy of Sciences, Novosibirsk 6300090, Russia.*
[3]*Medicinal Inorganic Chemistry Group, Department of Chemistry, University of British Columbia, 2036 Main Mall, Vancouver, BC V6T 1Z1, Canada.*
[4]*Faculty of Pharmaceutical Sciences, University of British Columbia, 2146 East Mall, Vancouver, BC V6T 1Z3, Canada.*
[+]*Present address: Department of Chemistry, Stanford University, Stanford, CA 94305, U. S. A.*

(Received: November 1, 2002; Accepted: January 2, 2003)

### ABSTRACT

The biological fate of a chelated vanadium source is investigated by *in vivo* spectroscopic methods to elucidate the chemical form in which the metal ion is accumulated. A pulsed electron paramagnetic resonance study of vanadyl ions in kidney tissue, taken from rats previously treated with bis(ethylmaltolato)oxovanadium(IV) (BEOV) in drinking water, is presented. A combined approach using stimulated echo (3-pulse) electron spin echo envelope modulation (ESEEM) and the two dimensional 4-pulse hyperfine sublevel correlation (HYSCORE) spectroscopies has shown that at least some of the $VO^{2+}$ ions are involved in the coordination with nitrogen-containing ligands. From the experimental spectra, a $^{14}N$ hyperfine coupling constant of 4.9 MHz and a quadrupole coupling constant of 0.6 ± 0.04 MHz were determined, consistent with amine coordination of the vanadyl ions. Study of VO-histidine model complexes allowed for a determination of the percentage of nitrogen-coordinated $VO^{2+}$ ions in the tissue sample that is found nitrogen-coordinated. By taking into account the bidentate nature of histidine coordination to $VO^{2+}$ ions, a more accurate determination of this value is reported. The biological fate of chelated versus free (i.e. salts)

* To whom correspondence should be addressed: S. A. D.: dikanov@uiuc.edu; C. O.: orvig@chem.ubc.ca.

vanadyl ion sources has been deduced by comparison to earlier reports. In contrast to its superior pharmacological efficacy over $VOSO_4$, BEOV shares a remarkably similar biological fate after uptake into kidney tissue.

### INTRODUCTION

The insulin-enhancing effects of vanadium complexes and salts have been well documented /1/. Despite a large volume of work, the mechanism(s) of action remain(s) to be delineated. Difficulties encountered in such studies arise from, among other factors, an incomplete understanding of the metabolism of chelated (i.e. coordination complexes) and "free" sources of vanadium (i.e. the common V(IV) salt vanadyl sulfate). The uptake and storage of vanadium species within specific tissues is of considerable interest both for the development of a complete metabolic model, and for detection of the putative "active species" (as yet undetected) ultimately responsible for insulin enhancement. The biodistribution and organ accumulation of exogenous vanadium species have been widely studied, particularly after the discovery of vanadium's insulin-enhancing effects. Bone, kidney and liver appear to be the primary organs of vanadium accumulation, irrespective of administered oxidation state and administration route /2-6/. Differences between organically chelated and inorganic sources appear to be minimal; the benchmark vanadium complex bis(maltolato)oxovanadium(IV) (BMOV, Figure 1) was preferentially accumulated in the same three tissues, although at concentrations 2-3 times higher than the inorganic salt $VOSO_4$ /5/. These similarities are likely due to an almost immediate dissociation of the chelating maltolato ligand in the bloodstream, as demonstrated in a recent report from our laboratories /7/. Earlier studies were limited due to the use of radioactive labeling, insensitive to both the coordination and the oxidation state. Thus, important information regarding the chemical form of accumulated vanadium is lacking, while such knowledge could shed some light on the structure of the active species.

Spectroscopic methods, however, can be used to gain insight into solid, solution and *in vivo* chemical structures. These methods must possess high selectivity to avoid saturation of the spectrum with unwanted features. For detection of $VO^{2+}$ *in vivo*, the paramagnetism of its complexes can be used to considerable

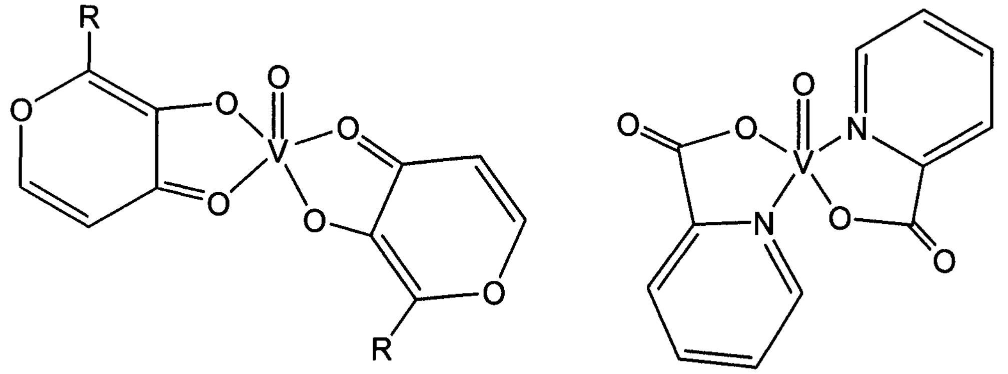

**Fig. 1:** Structure of bis(maltolato)oxovanadium(IV) (R = $CH_3$, BMOV) and bis(ethylmaltolato)oxovanadium(IV) (R = $CH_2CH_3$, BEOV) (left) and bis(picolinato)oxovanadium(IV), $VO(pic)_2$ (right).

advantage. Due to the strong electron spin magnetic moment, the relative magnetic susceptibility of an electron is 1800 times greater than that of the proton, therefore, detection limits in biological samples can reach the micromolar range. Additionally, paramagnetic resonance methods are naturally selective for paramagnetic species only; any interference from competing diamagnetic substances (e.g. V(V) complexes generated from *in vivo* oxidation of an administered V(IV) complex) is completely obviated. Thus, it would seem clear that EPR methods should be exceedingly useful in the study of the *in vivo* coordination structure of V(IV) species.

One of the first applications of pulsed EPR techniques to a true biological sample was reported by Fukui *et al.* in 1995 /8/. Wistar rats were treated by intravenous injection of $VOSO_4$ for 4 days, after which time they were sacrificed. Kidney and liver samples were then recovered and analyzed by continuous wave (cw) EPR and 2-pulse ESEEM spectroscopies. For both kidney and liver tissue, peaks identified with $^{14}N$ coupling and inner sphere protons from coordinated water molecules were observed. After assigning each peak, and processing using first order perturbation theory, the isotropic hyperfine $^{14}N$ couplings were compared to model VO(histidine) solutions, leading to the conclusion that the paramagnetic vanadyl centers had become ligated by one or more amines in these tissues.

While previous studies of *in vivo* vanadium distribution have been conducted /2,3,5/, little information was available regarding the chemical state, namely the oxidation and coordination states, of the metal ions. The application of pulsed EPR techniques permitted the acquisition of *in vivo* structural data on vanadyl ions within tissue samples, in turn potentially identifying a short-term storage form or a vanadyl-protein complex responsible for insulin enhancement. The $^{48}V$-$VOSO_4$ and $^{48}V$-BMOV study by our group revealed that, at least for BMOV, orally administered, chelated vanadyl sources were preferentially accumulated in bone, liver and kidney /5/. The Fukui *et al.* /8/ study was limited in that it used a first-generation (i.e. inorganic) anti-diabetic agent ($VOSO_4$) coupled with an intravenous route of administration.

We recently reported the *in vivo* coordination of vanadyl ions in bone mineral /9/, as well as a meticulous spectroscopic investigation of the $VO^{2+}$:triphosphate system as a model of the *in vivo* complex /10/. A wide variety of one (2, 3 and 4-pulse ESEEM) and two dimensional (HYSCORE) pulsed EPR techniques was used. These reports provided the first detailed study of the biological fate of chelated vanadyl sources in bone mineral, the primary site of vanadium accumulation. In this report, we present a spectroscopic study of vanadyl ions in BEOV-treated rat kidney samples. While earlier work reported on $VOSO_4$-treated rat kidney samples /8/, the work reported herein resolves several deficiencies in the previous study in that we present full 2 and 3-pulse one dimensional ESEEM spectra in addition to high resolution HYSCORE spectra for the complete delineation of the *in vivo* coordination of vanadyl ions in tissue. Further, the *in vivo* and model system spectra are presented herein as the Fourier transformation, eliminating signal distortion arising from application of other linear prediction analysis techniques such as the maximum entropy method (MEM). MEM processing produces very idealized, noiseless spectra from which information regarding the shape, width and intensity of ESEEM harmonics is lost; such spectra do not give any impression about the real quality of the frequency-domain data. Lastly, a more detailed analysis of the experimental spectra is provided, including comparisons to pulsed EPR studies of applicable chemical systems reported in the more recent literature.

### EXPERIMENTAL

Ethylmaltol (Pfizer) and vanadyl sulfate, $VOSO_4 \cdot 3H_2O$ (Aldrich), were used as received. EPR tubes (4 mm o.d., clear fused quartz) were obtained from Wilmad Glass. BEOV was synthesized according to established procedures and checked for purity by EA, MS (+LSIMS) and FT-IR /7/.

Male Wistar rats (University of British Columbia Animal Care Unit), weighing between 190 and 220 g, were acclimatized for 7 days, then divided into 4 groups: control (C), control-treated (CT), diabetic (D) and diabetic-treated (DT). Rats were rendered diabetic by injection of streptozotocin via the tail vein. Animals were kept in polycarbonate cages and allowed *ad libitum* access to food (Purina rat chow, Ralston Purina, St. Louis, MO) and tap water. The room was kept at 22 to 25 °C in a 12:12 h light:dark cycle. For treated animals (CT and DT groups), BEOV was dissolved in the drinking water, resulting in a typical dose of 0.26 - 0.29 mmol $kg^{-1}$ $d^{-1}$. The dosing was conducted for 6 weeks, at which time the animals were sacrificed by pentobarbital injection (100 mg $kg^{-1}$). Tissue samples (long bone, muscle, liver and kidney) were recovered and frozen at –20 °C.

Kidney tissue samples were cut into small pieces, quickly frozen in liquid $N_2$ and placed into EPR tubes previously flushed with Ar. Samples were sealed with parafilm, then re-frozen in dry ice and kept on dry ice or at –20 °C prior to spectroscopic study.

EPR spectra (both cw and pulsed) were obtained with a Bruker Elexsys E580 X-band spectrometer, interfaced with a Bruker ER035M teslameter for field calibration and a built-in Bruker frequency counter for microwave frequency measurement. Liquid He temperature studies were carried out using an Oxford Instruments CF 935 cryostat and an ITC 502 temperature controller. The samples were studied by several different ESEEM methods, the aforementioned two-pulse spin-echo sequence, a three-pulse stimulated echo experiment, and a two-dimensional hyperfine sublevel correlation (HYSCORE) four-pulse sequence. The three-pulse spectrum has the distinct advantage over the two-pulse spin echo experiment in that the modulation depth is much less dependent on the phase memory time. In addition, the spectrum is simplified due to the absence of nuclear sum and difference frequencies. The pulse sequence of $\pi/2$-$\tau$-$\pi/2$-T-$\pi/2$-$\tau$-*echo* leads to formation of a stimulated echo observed time $\tau$ after the third pulse. In the HYSCORE experiment ($\pi/2$-$\tau$-$\pi/2$-$t_1$-$\pi$-$t_2$-$\pi/2$-$\tau$-*echo*) /11/, the intensity of the stimulated echo after the fourth pulse is measured with $t_2$ and $t_1$ varied, and $\tau$ constant. This two-dimensional (2D) set of echo envelopes gives, after complex Fourier transformation, a 2D spectrum with equal resolution in each direction.

The basic advantage of the HYSCORE technique is the creation in 2D spectra of off-diagonal cross-peaks whose coordinates are nuclear frequencies from opposite electron spin manifolds. The cross-peaks significantly simplify the analysis of congested spectra by correlating and spreading out the nuclear frequencies /12/. In addition, the HYSCORE experiment separates overlapping peaks along a second dimension and enhances the signal-to-noise ratio through the application of a second Fourier transform /11,12/. Detailed consideration of the orientationally-disordered (i.e. powder or frozen solution) HYSCORE spectra for $I = ½$ and $I = 1$ nuclei is given elsewhere /13/.

### RESULTS AND DISCUSSION

Early in the study it was observed that no spectroscopic differences existed between the CT and DT tissue samples, and so all further discussion refers to spectra of tissue taken from CT rats treated with BEOV.

The first derivative of the field-sweep electron spin echo (FS-ESE) spectrum of BEOV-treated rat kidney is shown in Figure 2. The FS-ESE experiment collects echo intensity from a two-pulse sequence at various magnetic field values and is analogous to the adsorption mode of the EPR spectrum. The spectrum is typical for axial, magnetically dilute vanadyl ions, with spin Hamiltonian values $g_{\|} = 1.945 \pm 0.005$, $A_{\|} = 168 \pm 1 \times 10^{-4}$ cm$^{-1}$, $g_{\perp} = 1.98 \pm 0.01$ and $A_{\perp} = 58 \pm 1 \times 10^{-4}$ cm$^{-1}$. These values are appreciably different from those of BEOV ($g_x = 1.988$, $g_y = 1.976$, $g_z = 1.935$, $A_x = -60.5$, $A_y = -60.0$, $A_z = -170.0$) /7/, suggesting strongly a change has occurred in the coordination state of the vanadyl ions. Not surprisingly, no ligand superhyperfine coupling is observed.

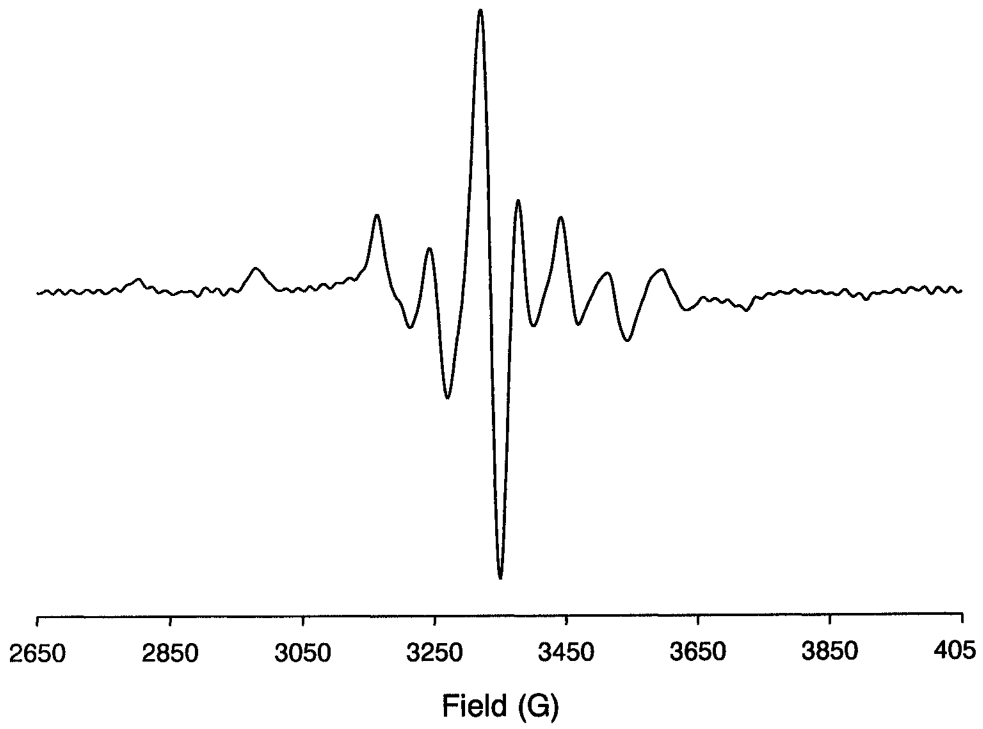

**Fig. 2:** First derivative field sweep ESE spectrum of rat kidney tissue, taken from an animal previously administered BEOV via drinking water ($T = 20$ K, $\nu = 9.3966$ GHz, $\tau = 200$ ns).

The ESEEM patterns were recorded on the different components of hyperfine structure. The stimulated ESEEM spectrum of the $VO^{2+}$ ions in BEOV-treated rat kidney taken at the central the $m_I^V = -\frac{1}{2}$ component (essentially orientation non-selective) of the FS-ESE spectrum is shown in Figure 3. The spectrum contains an intense line, at the proton Zeeman frequency of ~14 MHz, from the protons in the vicinity of the vanadyl ions. Additionally, two other peaks at 3.7 and 7.0 MHz are visible in the spectrum. Similar spectra were obtained at other points of the FS-ESE spectrum, corresponding to the $g_{\perp}$ and $g_{\|}$ components. The variations

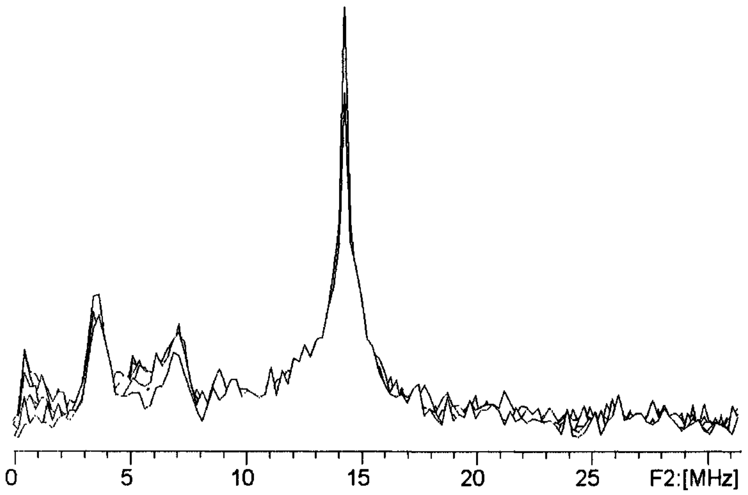

**Fig. 3:** Modulus FT spectrum of three-pulse ESEEM of BEOV-treated rat kidney (B = 333.3 mT, T = 30 K, ν = 9.3966 GHz).

of the frequencies of these two peaks at different points of the FS-ESE spectrum correspond well to twice the change in the value of the calculated $^{14}$N Zeeman frequency $\Delta(2|\nu_I|)$, indicating that the peaks are from two double quantum (dq) transitions of $^{14}$N nuclei from opposite electron spin manifolds /14/. In accordance with the analytical expression for dq-transitions the frequency of one peak increases while the other decreases as the strength of the magnetic field is increased (as shown in Equation 1).

$$\nu_{dq\pm} = 2\left[\left(\nu_I \pm \frac{A}{2}\right)^2 + K^2\left(3+\eta^2\right)\right]^{1/2} \quad (1)$$

$A$ = hyperfine coupling constant;
K = $e^2qQ/4h$, the quadrupole coupling constant;
$\eta$ = asymmetry parameter;
$\nu_I$ = $^{14}$N Zeeman frequency.

Assignment of these peaks was confirmed by the two dimensional HYSCORE experiment. The HYSCORE spectrum (Figure 4) shows cross-peaks in the (+,-) quadrant at (± 7.3, ∓ 3.6) MHz corresponding to the dq-dq correlations of the transitions observed in the 1D experiments. The traces of the sq-dq and sq-sq correlations (sq: single quantum) are also visible in the HYSCORE spectrum but are not analyzed due to their low intensity.

Using Equation 1, the hyperfine coupling constant $A$ and quadrupole parameter $K^2(3+\eta^2)$ can be determined from the two experimental frequencies 7.3 and 3.6 MHz /14/. Such a procedure yields $A$ = 4.9 MHz and $K^2(3+\eta^2)$ = 1.24 MHz$^2$ for $\nu_I$ = 1.0256 MHz. Assuming the asymmetry parameter is contained within $0 \leq \eta \leq 1$ one can obtain an estimate for K = 0.6 ± 0.04 MHz. The value of the hyperfine coupling constant determined from our data corresponds well to the earlier result of 5.0-5.2 MHz reported by Fukui *et al.*, obtained as a result of first-order analysis /8/.

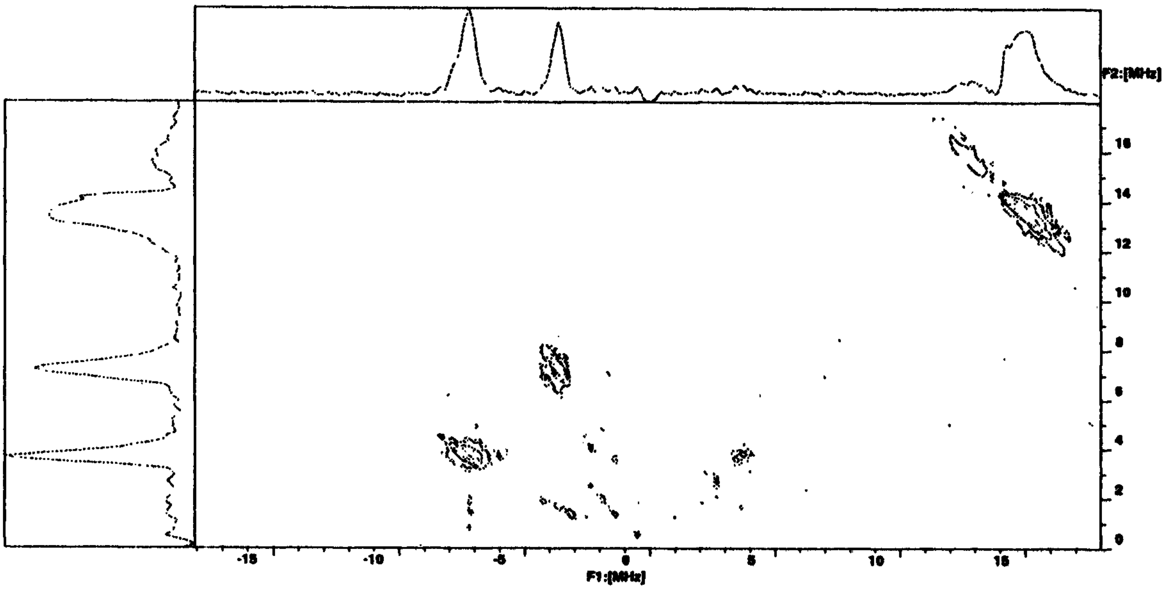

**Fig. 4:** HYSCORE spectrum of BEOV-treated rat kidney (B = 333.3 mT, T = 30 K, ν = 9.3966 GHz, τ = 200 ns, 256 x 256 points, step = 16 ns).

The values of the hyperfine and quadrupole couplings found from the ESEEM and HYSCORE spectra of $VO^{2+}$ ions in BEOV-treated rat kidney are consistent with vanadyl coordination by an amine nitrogen. As previously shown by Astashkin *et al.* /14/, and supported by later work (listed in detail in Table 1), the hyperfine couplings for equatorially coordinated simple amines have values of 4.5-5 MHz; such values are consistently smaller than those of coordinated imines. The quadrupole coupling constant of 0.6 MHz corresponds well to those previously reported for amine nitrogens coordinated to $VO^{2+}$[15,16], $Zn^{2+}$ or $Cu^{2+}$ /17-19/.

Isotropic $^{14}N$ coupling constants have been determined in a number of proteins where amine donation to the metal ion has been confirmed by other methods. Lysine coordination to $VO^{2+}$ ions has been observed in pyruvate kinase ($|A_{iso}|$ = 4.9 MHz) /15/, S-adenosylmethionine synthetase ($|A_{iso}|$ = 4.3-4.8 MHz) /20/ and F1 ATPase of spinach chloroplasts ($|A_{iso}|$ = 4.75 MHz) /21,22/. Lastly, direct axial coordination by $^{14}N$ can be ruled out, as several studies have demonstrated coupling constants in a much lower range, typically 2-3 MHz /23/.

**Table 1**

Magnitude of the isotropic $^{14}N$ hyperfine coupling constants of rat kidney samples previously treated with vanadium(IV) compounds.

| Complex | Administration Route | $\|A_{iso}\|$, MHz | Reference |
|---|---|---|---|
| BEOV | oral | 4.9 | This work |
| $VOSO_4$ | intravenous | 5.0-5.2 | 8 |
| $VOSO_4$ | intraperitoneal | ~4.8 | 25 |
| $VO(pic)_2$ | intraperitoneal | ~4.9 | 25 |

**Table 2**

Comparison of the isotropic coupling constant magnitudes for amine and imine equatorial $^{14}N$ donors to $VO^{2+}$ by ESEEM spectroscopy.

| Complex[a] | $^{14}N$ Donor Type | $\lvert A_{iso} \rvert$, MHz | Reference |
|---|---|---|---|
| $VO(gly)_2$ | Amine | 5.0 – 5.1 | 15,28 |
| $VO\text{-}NH_3$[b] | Amine | 4.7 | 29 |
| VO(edda) | Amine | 4.98 | 28 |
| $VO(acac)_2(py)$ | Imine | 5.7[c] | 14 |
| $VO(hfac)_2(L)$ | Imine | 7.1 (L = py) | 30 |
| | | 7.6 (L = ImH) | |
| $[VO(mim)_4Cl]^+$ | Imine | 6.4[d] | 31 |
| $VO(meox)_2$ | Imine | 6.0[d] | 32 |
| VO(salen) | Imine | 5.8 | 28 |
| $VO(Himac)_2(ImH)$ | Imine | 6.5 | 33 |

[a](ema = ethylmaltolate; acac = acetylacetonate; py = pyridine; gly = glycinate; edda = ethylenediaminediacetate; meox = 2-methylquinolin-8-olate; $H_2$salen = *N,N'*-bis(salicylidene)ethylenediamine; Himac = 4-imidazoleacetic acid; ImH = imidazole).

[b] on silica supported vanadium oxide.

[c] calculated by $A_{iso} = (2A_{\perp} + A_{\parallel})/3$.

[d] calculated by $A_{iso} = (A_x + A_y + A_z)/3$.

Fukui *et al.* used the 1:1 VO-histidine complex as a spectroscopic model of a nitrogen-coordinated $VO^{2+}$ complex in liver and kidney tissue, and as a reference for the quantitative determination of the percentage of the coordinated ions over the total $VO^{2+}$ ions in the sample /8/. They did not specify the structure of the complex and most likely assumed that the coordination of the histidine molecule occurred via the amine group only. However, detailed 2D ESEEM study of VO-imidazole and VO-histidine complexes has demonstrated that histidine is in fact a bidentate ligand coordinating $VO^{2+}$ ions via the α-amine and imine nitrogens /16/. ESEEM spectra of VO-histidine complexes are composed of the contributions of the coordinated amine nitrogen (with hyperfine coupling $A$ = 5.0 MHz, and a quadrupole coupling constant K = 0.58 ± 0.02 MHz) and the coordinated imine nitrogen of the imidazole ($A$ = 6.3 MHz, and K = 1.02 ± 0.07 MHz). The special peculiarity of this system is that the intensity of the lines from the amine nitrogen in two- and three-pulse spectra is significantly larger than from the imine nitrogen /16/; these differences were not recognized in the MEM spectra reported by Fukui *et al.* /8/. Nevertheless, imine nitrogens observably affect the ESEEM spectra of VO-histidine complexes and should be considered in the quantitative analysis of the percentage of the nitrogen-coordinated $VO^{2+}$ in the sample.

Figures 5a, 5c and 5e show time-domain two-pulse ESEEM patterns of BEOV-treated rat kidney, $VO(his)_2$ (his = histidine) as well as the $^{15}N$-labeled (at the imine nitrogen of the imidazole moiety) complex, $VO(1\text{-}^{15}N\text{-}his)_2$, respectively. It is clear that the amplitude of the low frequency signals (due to nitrogen

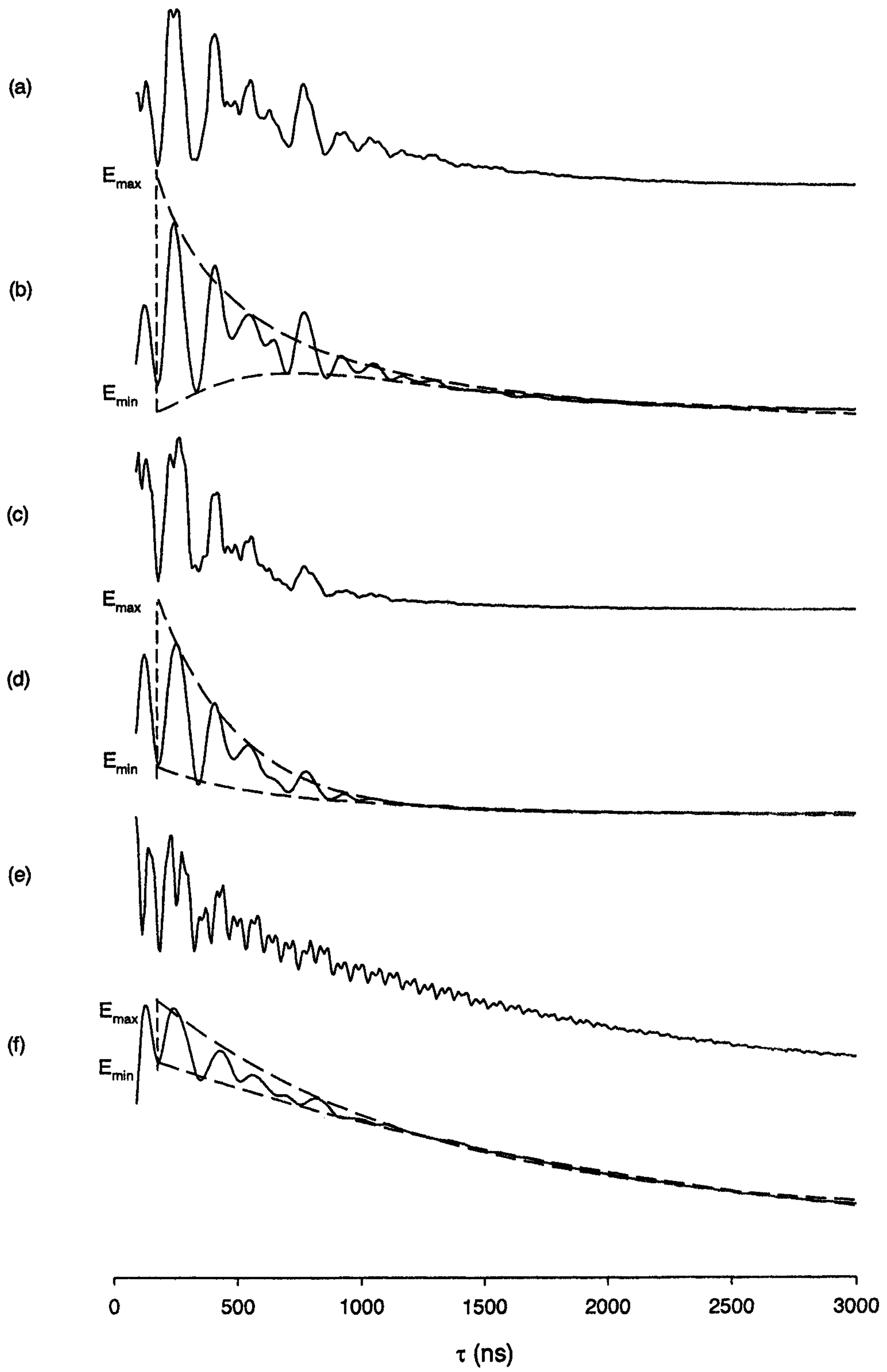

**Fig. 5:** Two-pulse time domain patterns of (a) $VO(His)_2$ (ν = 9.75 GHz, B = 346.8 mT), (b) previous spectrum after filtration of high frequency contributions and fit of modulation decays, (c) $VO(1\text{-}^{15}N\text{-}His)_2$ (ν = 9.74 GHz, B = 345.3 mT), (d) after filtration and fitting, (e) BEOV-treated rat kidney (ν = 9.39 GHz, B = 333.3 mT) (f) after filtration and fitting.

nuclei) relative to the proton frequencies is significantly smaller for the *in vivo* sample than for the model complexes, thus indicating that only part of the $VO^{2+}$ present in the biological sample is coordinated to nitrogen atoms, or that only one nitrogen atom is coordinating the $VO^{2+}$ *in vivo* instead of the two in the model complexes. This difference is also manifested in the corresponding modulus FT plots shown in Figure 6.

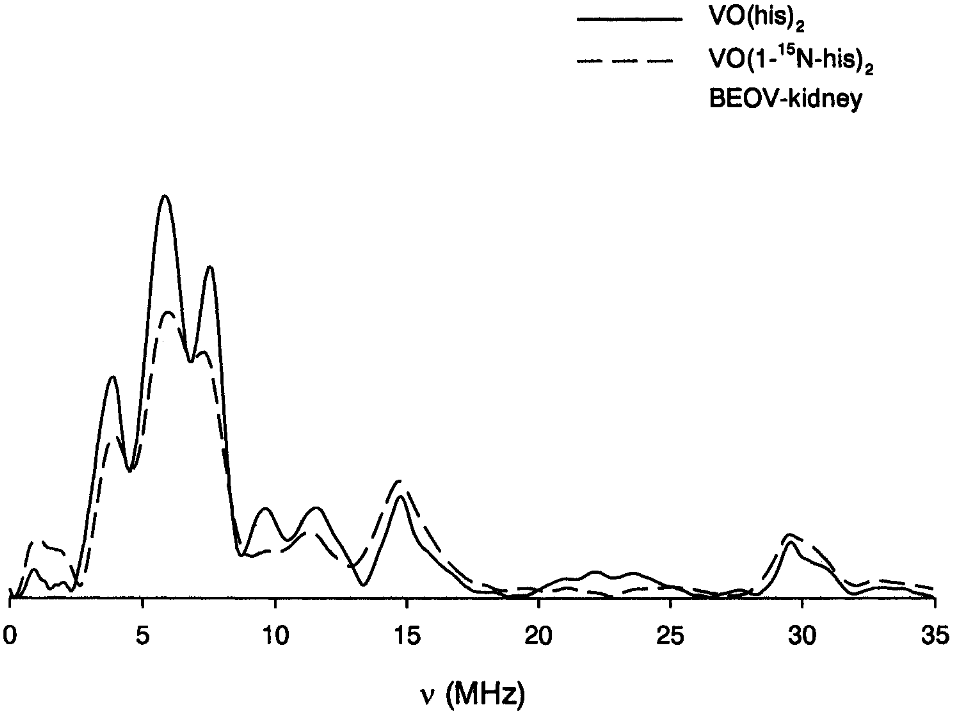

**Fig. 6:** Modulus FT two-pulse spectra of (a) $VO(His)_2$, (b) $VO(1\text{-}^{15}N\text{-}His)_2$ and (c) BEOV-treated rat kidney (parameters identical to those listed in Fig. 5).

Contained within each frequency-domain spectrum in Figure 6 is a triplet of lines at frequencies <8 MHz; each triplet includes two dq transitions and a new intense line corresponding to the combination of the sq transitions, $\nu_{sq+}^{(1,2)} + \nu_{sq-}^{(2,1)}$. The frequency of this peak, to a first-order approximation, is equal to the nitrogen hyperfine coupling constant. Comparison of the spectra of the natural abundance and $^{15}N$-labeled $VO(his)_2$ complexes reveals clear differences between them in the relative intensity of the triplet components as well as in the lineshapes of two lower intensity lines between 10 and 12 MHz. These differences reflect the additional contribution of the $^{14}N$ imine nitrogens to the ESEEM pattern. Upon $^{15}N$ substitution, this contribution is eliminated, producing only very minor influences on the ESEEM spectra, but at different frequency ranges compared to $^{14}N$.[16] As a result, the $VO(1\text{-}^{15}N\text{-}his)_2$ complex is a more correct model for the quantitative determination of the percentage of nitrogen-coordinated $VO^{2+}$ ions.

The percentage of the total $VO^{2+}$ concentration involved in nitrogen coordination can be estimated from the depth of nitrogen ESEEM in the time-domain spectra. However, the ESEEM spectra in Figure 6 show the presence of peaks from protons with matrix frequencies of ~14 and 28 MHz, which are near exact multiples of the dq frequencies of 3.7 and 7.0 MHz. Such concurrences might affect the modulation depth of the nitrogen signals due to coincidences of the modulation minima from the two types of nuclei ($^{14}N$ and $^{1}H$), especially at the initial part of the ESEEM patterns where the proton modulation is deep. Filtration of the high frequencies (>12 MHz) from the two-pulse ESEEM patterns of Figures 5a, 5c and 5e yields the patterns shown in 5b, 5d and 5f, respectively, eliminating modulation depth distortions caused by the presence of proton signals in the ESEEM patterns. The decay of the signal in each of these patterns has been fitted with an arbitrary exponential decay curve, for the estimation of the initial modulation depth for each sample (*vide infra*).

If we define $V_N(\tau)$ as the normalized ESEEM with $V_N(0) = 1$ from one amine nitrogen, then the experimental echo intensity is described by Equation 2.

$$E(\tau) = N\,D(\tau)\,V_N(\tau) \tag{2}$$

$N$ is the numerical coefficient determined from the real scale of the experimental echo intensity, unique for each sample and $D(\tau)$ is the function describing the relaxation decay of the echo signal. For the $VO(his)_2$ complex, where two equivalent nitrogens contribute to the ESEEM, the equation is slightly different (Equation 3) /24/.

$$E(\tau) = N\,D(\tau)\,[V_N(\tau)]^2 \tag{3}$$

One can also determine $[E(\tau)]_{min}$ and $[E(\tau)]_{max}$ as envelopes drawn through the points of minimum and maximum echo amplitude (Figure 5b, 5d and 5f, dashed lines). Then, for any $\tau$, the modulation depth in the experimental curves from one nitrogen nucleus can by described by the ratio in Equation 4, or, as in Equation 3, for two nitrogens (Equation 5).

$$d_1 = \frac{E_{min}}{E_{max}} = \frac{[V_N(\tau)]_{min}}{[V_N(\tau)]_{max}} \tag{4}$$

$$d_2 = \frac{E_{min}}{E_{max}} = \left\{\frac{[V_N(\tau)]_{min}}{[V_N(\tau)]_{max}}\right\}^2 \tag{5}$$

If it is assumed that in the *in vivo* sample only part of the $VO^{2+}$ ions are nitrogen-coordinated, then each normalized intensity is described by Equation 6 ($x$ is the percentage of the vanadyl ions in the sample which are nitrogen-coordinated).

$$E(\tau) = N\,D(\tau)\left[(1-x) + xV_N(\tau)\right] \tag{6}$$

The modulation depth in this case is described by Equation 7.

$$d = \frac{E_{min}}{E_{max}} = \frac{(1-x) + x[V_N(\tau)]_{min}}{(1-x) + x[V_N(\tau)]_{max}} \tag{7}$$

If we introduce the related parameter λ, or modulation amplitude, we can obtain Equation 8.

$$\lambda = 1 - d = \frac{x([V_N(\tau)]_{max} - [V_N(\tau)]_{min})}{(1-x) + x[V_N(\tau)]_{max}} \tag{8}$$

By expanding upon the binomial fraction shown above we obtain the modulation amplitude relative to $d_1$ (Equation 9).

$$\lambda = 1 - d = \left(\frac{x[V_N(\tau)]_{max}}{(1-x)+x[V_N(\tau)]_{max}}\right)\left(1 - \frac{[V_N(\tau)]_{min}}{[V_N(\tau)]_{max}}\right) = \left(\frac{x[V_N(\tau)]_{max}}{(1-x)+x[V_N(\tau)]_{max}}\right)(1-d_1);\ \lambda_1 = 1 - d_1 \tag{9}$$

By solving for x, we obtain Equation 10.

$$x = \frac{\lambda}{\{\lambda + [V_N(\tau)]_{max}(\lambda_1 - \lambda)\}} \tag{10}$$

This analysis shows that if only a percentage of the total $VO^{2+}$ ions present in the sample is coordinated to nitrogen, then the modulation amplitude λ = 1-d is determined by the coefficient $\frac{x[V_N(\tau)]_{max}}{(1-x)+x[V_N(\tau)]_{max}}$ compared to $(1-d_1)$ with 100% coordination. Analysis of this coefficient shows that it approaches x when $[V_N(\tau)]_{max} \to 1$.

Because the exact function $V_N(\tau)_{max}$ is unknown, an estimate of the value of x using available experimental data must be employed. Equations 1-10 detailed above provide a method by which reasonably accurate estimates can be obtained from experimental spectra. In orientationally-disordered samples, the modulation depth is inversely proportional to the interpulse time; the modulations decrease with increases in the time between the two pulses of the ESEEM experiment. Therefore, the most accurate estimate of x can be obtained from d and $d_1$ measured at the shortest possible times τ of the experimental envelopes where $V_N(\tau)_{max}$ is close to 1.

The measurement of modulation depth was performed on two-pulse ESEEM patterns obtained (after filtration of the proton frequencies, *vide supra*) for the *in vivo* sample and two model complexes ($VO(his)_2$ and $VO(1\text{-}^{15}N\text{-}his)_2$) at the same time, as indicated in each of Figures 5b, 5d and 5f. The depths obtained were d = 0.70 (*in vivo*), $d_2$ = 0.25 ($VO(1\text{-}^{15}N\text{-}his)_2$), $d_2$ = 0.11 ($VO(his)_2$). All parameters were determined with an accuracy of ± 0.02. The latter two parameters allowed for the calculation to find $d_1$ = 0.50 ($VO(1\text{-}^{15}N\text{-}his)_2$) and $d_1$ = 0.33 ($VO(his)_2$). Substitution of the d and $d_1$ values into Equation 10 yields (assuming $V_N(\tau)_{max}$ ~ 1) minimum x values of 0.60 and 0.45 for the labeled and unlabeled samples, respectively. The value of 0.45 is close to the previously determined value of 0.5 reported by Fukui *et al.* for the non-labeled sample /8/. These values would progressively increase as the value of $V_N(\tau)_{max}$ decreases. If, for instance, $V_N(\tau)_{max}$ was found to be ~ 0.8, then the estimate for x gives 0.67 and 0.50. Thus, by using the unlabeled $VO(his)_2$ complex as a basis for a quantitative comparison of the nitrogen-coordinated vanadyl ions in the biological sample, Fukui *et al.* underestimated this value by 10-17%. Our comparison, however, was made with BEOV-treated tissue, obtained from rats subjected to a profoundly different dosing procedure; we discuss these differences in the next section.

### *In Vivo* Accumulation of Vanadium(IV).

This work provides intriguing comparisons to earlier reported *in vivo* data on the bioaccumulation of vanadyl species. We have shown in the previous section that oral administration of BEOV and subsequent bioaccumulation in kidney tissue results in at least partial degradation of the complex; as much as 67% of the detected ions are found in the nitrogen-coordinated form. This percentage is in fact *higher* than that previously determined for intravenously-administered $VOSO_4$ /8/, indicating that the oral administration pathway subjects vanadyl complexes (at least those studied to date) to significant biotransformation activities that result in loss of ligand(s) prior to organ accumulation. This conclusion is supported by our earlier study of vanadyl ions (from BEOV) in bone mineral which also demonstrated complex degradation prior to accumulation in the bone matrix /9/.

Fukui *et al.* reported 2-pulse ESEEM spectra of kidney samples, taken from rats previously treated (via intraperitoneal injection) with bis(picolinato)oxovanadium(IV) ($VO(pic)_2$) (Figure 1) and $VOSO_4$ /25/. Contained within the ESEEM spectra of $VO(pic)_2$ samples was a weak signal corresponding to a coordinated imine nitrogen, suggestive therefore of the presence of one of the original picolinato ligands in the first coordination sphere of the paramagnetic ions. We do not detect imine coordination at all in our study, which would seem to corroborate the observations of Fukui *et al.* /25/. Their observation, however, does raise some interesting mechanistic considerations in light of our results. In the current work, BEOV was administered via drinking water to rats, therefore the paramagnetic vanadyl ions must have been absorbed from the gastrointestinal tract, transported to the kidneys via the bloodstream, and ultimately absorbed again into the renal tissue. Administration by intraperitoneal injection, however, would serve to bypass most if not all but the final process. The $VO(pic)_2$ found in the kidney tissue would therefore bypass several potential sites of biotransformation and hence potentially arrive in the tissue as the intact complex. Current data in our group and others indicates that the BMOV family of complexes is susceptible to degradation via reaction with serum proteins such as apo-transferrin and albumin /26/. Since the thermodynamic stability of $VO(pic)_2$ is actually several orders of magnitude *lower* than that of BEOV ($\log \beta_2 = 11.99$ for $VO(pic)_2$[27] versus 16.43 for BEOV) /7/, it is implausible that $VO(pic)_2$ would survive as the intact complex in the bloodstream. Additionally, greater than 60% of the vanadyl ions present in the kidney sample (from the original BEOV source) is found in the amine-coordinated form; considering the greater thermodynamic stability of BEOV over $VO(pic)_2$ and the vigorous route of administration (oral versus intraperitoneal injection), it seems likely, all conditions being equal, that an orally-administered $VO(pic)_2$ sample would undergo a much greater degree of biotransformation than that indicated in the Fukui *et al.* study. Thus, the possibility that the imine-amine coordinated vanadyl species (i.e. the original $VO(pic)_2$ complex) detected in the Fukui *et al.* study is in some way responsible for the augmented anti-diabetic effects is remote. Further, since the kidneys themselves have little involvement in carbohydrate and lipid metabolism, it is in fact much more likely that the amine-coordinated species detected in both studies is an end-product of *in vivo* transformation of the administered complex, downstream of the anti-diabetic effect(s).

### CONCLUSIONS

By utilizing pulsed EPR techniques, we have demonstrated that the biological fate of chelated vanadium sources such as BMOV is remarkably similar to that of free sources (e.g. $VOSO_4$) in rat kidney tissue. Administered $VO^{2+}$ from either BMOV or $VOSO_4$ becomes nitrogen-ligated, in approximately the same proportion. A detailed consideration of the time-domain spectra of the *in vivo* sample and two model complexes shows that modulation depths can be used to obtain reasonable estimates of the percentage of the total detected paramagnetic species interacting with the nucleus of interest. Such an analysis requires, however, isolation of the undistorted modulation depth arising from the nucleus under study. We have presented an improved method for determining the relative proportion of a particular species, and shown that a previously reported method underestimates this value. With the biological fate in the kidney of both chelated and free vanadium sources remarkably similar, the superior insulin-enhancing activity of chelated sources such as BMOV must be a result of either a key difference in some other metabolic pathway, or merely increased gastrointestinal absorption via oral administration. Due to the predominantly excretory role of the kidneys, it is likely that this difference(s) occurs prior to accumulation of administered vanadium in these organs.

### ACKNOWLEDGEMENTS

We thank Kinetek Pharmaceuticals Inc., the Canadian Institutes of Health Research, the Science Council of British Columbia (GREAT program, B.D.L.), and the Natural Sciences and Engineering Research Council of Canada for support of this research. This work was supported by NIH grants RR01811 to the Illinois EPR Research Center and GM62954 to S.A.D.

### REFERENCES

1. K.H. Thompson, J.H. McNeill and C. Orvig, *Chem. Rev.*, **99**, 2561 (1999).
2. M.A. Al-Bayati, O.G. Raabe, S.N. Giri and J.B. Knaak, *J. Am. Coll. Toxicol.*, **10**, 233 (1991).
3. R.D.R. Parker and R.P. Sharma, *J. Environ. Pathol. Toxicol.*, **2**, 235 (1978).
4. E. Sabbioni, E. Marafante, L. Alantini, L. Ubertalli and C. Birattari, *Bioinorg. Chem.*, **8**, 503 (1978).
5. I.A. Steyawati, K.H. Thompson, V.G. Yuen, Y. Sun, M. Battell, D.M. Lyster, C. Vo, T.J. Ruth, S. Zeisler, J.H. McNeill and C. Orvig, *J. Appl. Physiol.*, **84**, 569 (1998).
6. T.B. Wiegmann, H.D. Day and R.V. Patak, *J. Toxicol. Environ. Health*, **10**, 233 (1982).
7. K.H. Thompson, B.D. Liboiron, K.D.D. Bellman, I.A. Setyawati, B.O. Patrick, V. Karunaratne, G. Rawji, J. Wheeler, K. Sutton, S. Bhanot, C. Cassidy, J.H. McNeill, V.G. Yuen and C. Orvig, *J. Biol. Inorg. Chem.*, **8**, 66 (2003).
8. K. Fukui, H. Ohya-Nishiguchi, M. Nakai, H. Sakurai and H. Kamada, *FEBS Lett.*, **368**, 31 (1995).
9. S.A. Dikanov, B. Liboiron, K.H. Thompson, E. Vera, V.G. Yuen, J.H. McNeill and C. Orvig, *J. Am. Chem. Soc.*, **121**, 11004 (1999).

10. S.A. Dikanov, B.D. Liboiron and C. Orvig, *J. Am. Chem. Soc.*, **124**, 2969 (2002).
11. P. Höfer, A. Grupp, H. Nebenführ and M.M. Mehring, *Chem. Phys. Lett.*, **132**, 279 (1986).
12. S.A. Dikanov, A.M. Tyryshkin and M.K. Bowman, *J. Magn. Reson.*, **144**, 228 (2000).
13. S.A. Dikanov, in: *New Advances in Analytical Chemistry*, Attar-ur-Rahman (Ed.), Gordon and Breach, Amsterdam, 2000; p. 523.
14. A.V. Astashkin, S.A. Dikanov and Y.D. Tsvetkov, *J. Struct. Chem.*, **26**, 363 (1985).
15. P.A. Tipton, J. McCracken, J.B. Cornellius and J. Peisach, *Biochemistry*, **28**, 5720 (1988).
16. S.A. Dikanov, R.I. Samoilova, J.A. Smieja and M.K. Bowman, *J. Am. Chem. Soc.*, **117**, 10579 (1995).
17. C.I.H. Ashbi, W.F. Paton and T. Brown, *J. Am. Chem. Soc.*, **102**, 2990 (1980).
18. C.A. MacDowell and A. Naito, *J. Magn. Reson.*, **45**, 205 (1981).
19. C.A. MacDowell, A. Naito, D.L. Sastry, Y.U. Cui, K. Sha and S.X. Yu, *J. Mol. Struct.*, **195**, 361 (1989).
20. C. Zhang, G.D. Markham and R. LoBrutto, *Biochemistry*, **32**, 9866 (1993).
21. A.L.P. Houseman, L. Morgan, R. LoBrutto and W.D. Frasch, *Biochemistry*, **33**, 4910 (1994).
22. C. Buy, T. Matsui, S. Rianambininstoa, C. Sigalat, G. Girault and J.L. Zimmerman, *Biochemistry*, **35**, 14281 (1996).
23. Y. Deligiannakis, M. Louloudi and N. Hadjiliadis, *Coord. Chem. Rev.*, **204**, 1 (2000).
24. Equation 3 assumes that ESEEM from two nuclei in a disordered sample is calculated as $V_2 = \langle V_1 \rangle^2$. This is an approximate expression. An exact formula in the case of magnetically equivalent nuclei has the form $V_2 = \langle (V_1)^2 \rangle$. However, it has been shown (see for example, S.A. Dikanov and Yu.D. Tsvetkov, *Electron Spin Echo Envelope Modulation (ESEEM) Spectroscopy*, CRC, Boca Raton, 1992; p. 101-104) that both methods of calculation give practically the same result for weak anisotropic hyperfine interactions when $T/\nu_I < 0.5$. This relation is well satisfied for $^{14}N$ coordination to $VO^{2+}$. Therefore, we used Equation 3 to estimate the $^{14}N$ modulation amplitude in the model complexes because the expression $V_2 = \langle (V_1)^2 \rangle$ does not allow such an estimate.
25. K. Fukui, Y. Fujisawa, H. Ohya-Nishiguchi, H. Kamada and H. Sakurai, *J. Inorg. Biochem.*, **77**, 215 (1999).
26. B.D. Liboiron, E. Lam, G.R. Hanson, N. Aebischer and C. Orvig (in preparation).
27. T. Duma and R. Hancock, *J. Coord. Chem.*, **31**, 135 (1994).
28. K. Fukui, H. Ohya-Nishiguchi and H. Kamada, *Inorg. Chem.*, **36**, 5518 (1997).
29. S. Cosgrove-Larsen and D.J. Singel, *J. Phys. Chem.*, **96**, 9007 (1992).
30. S.S. Eaton, J. Dubach, K.M. More, G.R. Eaton, G. Thurman and D.R. Ambruso, *J. Biol. Chem.*, **264**, 4776 (1989).
31. E.J. Reijerse, J. Shane, E. de Boer and D. Collison, in: *Electron Magnetic Resonance of Disordered Systems*, N.D. Yordanov (Ed.), World Scientific, Singapore, 1989, p. 189.
32. E.J. Reijerse, J. Shane, E. de Boer, P. Höfer and D. Collison, in: *Electron Magnetic Resonance of Disordered Systems,* N.D. Yordanov (Ed.), World Scientific, Singapore, 1991; p. 253.
33. K. Fukui, H. Ohya-Nishiguchi and H. Kamada, *Inorg. Chem.*, **38**, 2326 (1998).

